# An Active-Learning Framework for Educating Medical Students on SARS-CoV-2 Variants and COVID-19 Epidemiology

**DOI:** 10.12688/mep.20540.1

**Published:** 2024-11-15

**Authors:** Samiksha Prasad, Amanda J Chase

**Affiliations:** 1 Nova Southeastern University Dr Kiran C Patel College of Allopathic Medicine, Fort Lauderdale, Florida, USA

**Keywords:** COVID-19, SARS-CoV-2 Variants, Team-Based Learning, Coronaviruses, Epidemiology, Infectious Diseases, Medical Education, Active Learning

## Abstract

**Background:**

The emergence of multiple Severe Acute Respiratory Syndrome-Coronavirus-2 (SARS-CoV-2) variants presented an escalated risk to public health globally and prompted epidemiologic monitoring and classification. Health professionals are vital for patient education regarding Coronavirus Disease 2019 (COVID-19), discussing patient concerns, and providing guidance. Students enrolled in professional healthcare programs benefit from being adept with the evolution and spread of SARS-CoV-2 variants, and a team-based learning module can be helpful for applying foundational concepts to clinical problems.

**Methods:**

This team-based learning (TBL) framework was developed in response to the COVID-19 pandemic and the emergence of viral variants. It was placed at the end of a hematology block within the first semester of year one of the medical school during the academic years 2021–2022. It consists of a 7-question readiness assurance process and a four-question application exercise.

**Results:**

The average score increased from 58.8% (iRAT) to 85.9% (tRAT) (n=104). The post-session survey data showed an increase in students’ understanding of the classification of COVID-19 variants and the role of genetic mutations in viral pathogenesis. Qualitative data yielded positive feedback for the session, notably in students' ability to interpret phylogenetic trees and understand the role of variants.

**Conclusions:**

This TBL framework cultivates higher-order thinking skills among medical students and effectively integrates virology, epidemiology, and pathology. Additionally, it provides a framework for developing a robust and up-to-date platform for the discussion of novel variants of COVID-19 or other infectious diseases.

## Introduction

Coronaviruses (CoVs) are a diverse and large family of enveloped, zoonotic, single-stranded RNA viruses. There are four genera of CoVs (alpha, beta, gamma, and delta), with alpha and beta having the ability to cause disease in humans by crossing animal-human barriers and becoming human pathogens ( Mistry *et al.*, 2022; Zhu *et al.*, 2020). Severe Acute Respiratory Syndrome-Coronavirus-2 (SARS-CoV-2) is the latest coronavirus family member and the causative agent of the Coronavirus Disease 2019 (COVID-19) pandemic, which started in 2019 and lasted until 2023 ( Mackenzie & Smith, 2020; Mistry *et al.*, 2022). The widespread transmission of SARS-CoV-2 across the globe has resulted in the continuous genetic evolution of the virus at a high rate. This is attributed to changes in the genetic code, known as genetic mutations, which occur during the replication of the viral genome ( Plante *et al.*, 2021). These mutations can sometimes lead to the emergence of new viral variants. Some variants emerge and disappear, whereas others persist ( Plante *et al.*, 2021). Given the impact of variants on public health due to the continuous evolution of SARS-CoV-2, the Centers for Disease Control and Prevention classifies variants based on their traits and prevalence in the United States as: (i) variants being monitored (VBM), (ii) variant of interest (VOI), (iii) variant of concern (VOC), and (iv) variant of high consequence (VOHC) ( Centers for Disease Control and Prevention, 2023).

Recent increases in the incidence of vaccine-preventable illnesses and vaccine hesitancy are considered a consequence of fast-paced, non-validated, and social-media-based misinformation ( De Figueiredo *et al.*, 2020). Evidence for a significant correlation between vaccine hesitancy and misinformation has become even more pronounced during the COVID-19 pandemic ( Kreps *et al.*, 2021; Neely *et al.*, 2022). A recent study by the Surgeon General on combating health misinformation urges educators and educational institutions to expand and deepen evidence-based programs that help students become resilient to false information ( Office of the Surgeon General, 2021). Health professionals can and should play an important role in mitigating mistrust of COVID-19 vaccines. Specific approaches, including motivational interviewing, have been proposed for clinicians to address the mistrust of COVID-19 vaccines among minority groups. Another recommendation is to leverage trusted community leaders or organizations to engage minority populations in public health campaigns that promote education and awareness ( Opel *et al.*, 2021). A recent study by Fisher *et al.* found that a COVID-19 vaccine recommendation by a healthcare practitioner reduced COVID-19 vaccine hesitancy among patients ( Fisher *et al.*, 2023).

Traditionally, curricula in health professions have focused on the pathogen and disease state with little emphasis on viral epidemiology ( Kushner & Pekosz, 2021; Shahmanesh *et al.*, 2020). This study aimed to promote an understanding of the transmission and emergence dynamics of viral variants among future healthcare providers through an engaging framework. This addresses the need for training healthcare providers to understand viral variants, and disease prevention is paramount to the provision of evidence-based care and represents these topics in health profession curricula ( Nayahangan *et al.*, 2021).

A team-based, active learning approach was chosen to critically analyze the transmission dynamics and epidemiological characteristics of SARS-CoV-2 variants of concern. Through a series of exercises that involved individual work, teamwork, and quick feedback, this small group-active learning instructional approach gave students the opportunity to apply conceptual information. This format allows students to learn collaboratively, apply their existing knowledge, and understand concepts through real-time research methods. The team collaboration approach facilitates the application of higher-order concepts, promotes learner engagement, and fosters positive learner attitudes ( Balwan *et al.*, 2015; Beckler & Chase, 2021; Parmelee *et al.*, 2012). Team-based learning (TBL) has become an increasingly popular strategy for engaging learners in undergraduate medical education.

## Methods

### Curricular context

This TBL module was developed as part of the Hematology Block, a 4-week block designed for first-year allopathic medical students that falls during the first semester. Prior to this block, students took a 13-week fundamentals course to acquire foundational knowledge in the sciences basic to medicine. This module involves the application of the mechanisms through which different viral variants of SARS-CoV-2 can emerge. The focus of the content included the interpretation of SARS-CoV-2 viral variants and the impact of immunization, travel, and epidemiology on the emergence and spread of variants. This TBL builds upon fundamental concepts in infectious diseases and genetic evolution and connects real-world scenarios with the interpretation of evidence-based medicine. We delivered this TBL module to first-year medical students in 2021 and 2022. Facilitators were provided with a facilitator’s guide as a resource for delivering the module. The educational objectives of the session were (i) to describe the development of viral variants; (ii) to understand the role of immunization, travel, and epidemiology in the emergence of viral variants; (iii) to compare and contrast SARS-CoV-2 variants of concern; and (iv) to interpret a phylogenetic tree of SARS-CoV-2 variants.

### Team formation

At the beginning of each semester, the first-year medical student cohort (51 students) was divided into 10 TBL teams of 5-6 members each. The teams were divided into equal groups based on randomized numbering. This ensured diversity, a comparable range of expertise, and previous training/education across all groups.

### Advance preparation resources

Learning objectives and a pre-reading assignment were made available to the students 2 weeks prior to the educational session. The learning objectives provided the students with guidelines to prepare appropriate content and depth for the session. As preparation materials for the TBL module, appropriate resources were recommended for background information on SARS-CoV-2 lineages and variants, their classifications, and definitions by the Centers for Disease Control and Prevention (CDC). Supplementary to this, a comprehensive list of recommended reading materials comprised of videos and peer-reviewed content was provided.

### Readiness assurance process

To ensure that students were ready for higher-order activities within the session, an individual quiz was given at the beginning of the session. The quiz comprised seven first- and second-order multiple-choice questions (MCQs) that were written to assess the students’ readiness and understanding of the pre-assigned readings. Students were provided with a total of 10 minutes to complete the 7-question closed-resource test. The Canvas with Respondus LockDown Browser electronic educational platform (Instructure Inc., Salt Lake City, Utah) was used to deliver the quiz, and scores were not immediately provided to the students after the completion of the quiz. Following the individual quiz, the students took a team quiz that contained the same multiple-choice questions as the individual quiz. A team quiz was administered through the InteDashboard educational platform (CognaLearn Pte. Ltd., Singapore) to provide instant feedback on each question's correct response. The team quiz was a closed resource, and teams were given 15 min to work together on the MCQs. Full credit was assigned for each question if the teams selected the accurate answer on the first attempt; otherwise, there was a progressive deduction of points for each incorrect answer selected. This approach provided the team with additional attempts to answer each question accurately. Following the team quiz, instructor clarification was provided as a large-group discussion to address any student questions or doubts that had not been resolved by the team.

### Team application activities

Team application activities were administered to student teams after the individual and team readiness assurance tests. Application-based activities were released sequentially, and team progression was monitored using the InteDashboard educational platform. Application activities were sourced from the primary literature to cultivate the interpretation of research data and their application to patient care. The first activity involved the team interpretation of a phylogenetic tree that demonstrated the evolution of SARS-CoV-2 variants. The second activity involved team analysis of dose-response curves of neutralization of SARS-CoV-2 variants by four monoclonal antibodies. ^15^ Following each activity, the facilitators called for reporting of answers from each team.

### Post TBL survey

The Post-TBL Survey was disseminated post-session and consisted of 11 items. Ten items gauge student perceptions of engagement within the session, and two open-ended prompts asked students to reflect on the TBL and COVID-19 pandemic. The items on session engagement were based on the Assessment Student Perspective of Engagement in Class Tool ( Wiggins *et al.*, 2017).

## Results

Overall, 102 first-year medical students participated in TBL sessions over two consecutive years ( Chase & Prasad, 2024). In 2021, the averages for iRAT and tRAT were 58.8% and 85.9% respectively. In 2022, the averages were 59.1% and 87.8% for iRAT and tRAT, respectively. While the team application activities were not graded, students had to be present and engaged to receive a grade for the TBL session. Comparable and lively discussions and response rates were reported across both years of delivery of the team application activities.

**Figure 1.**
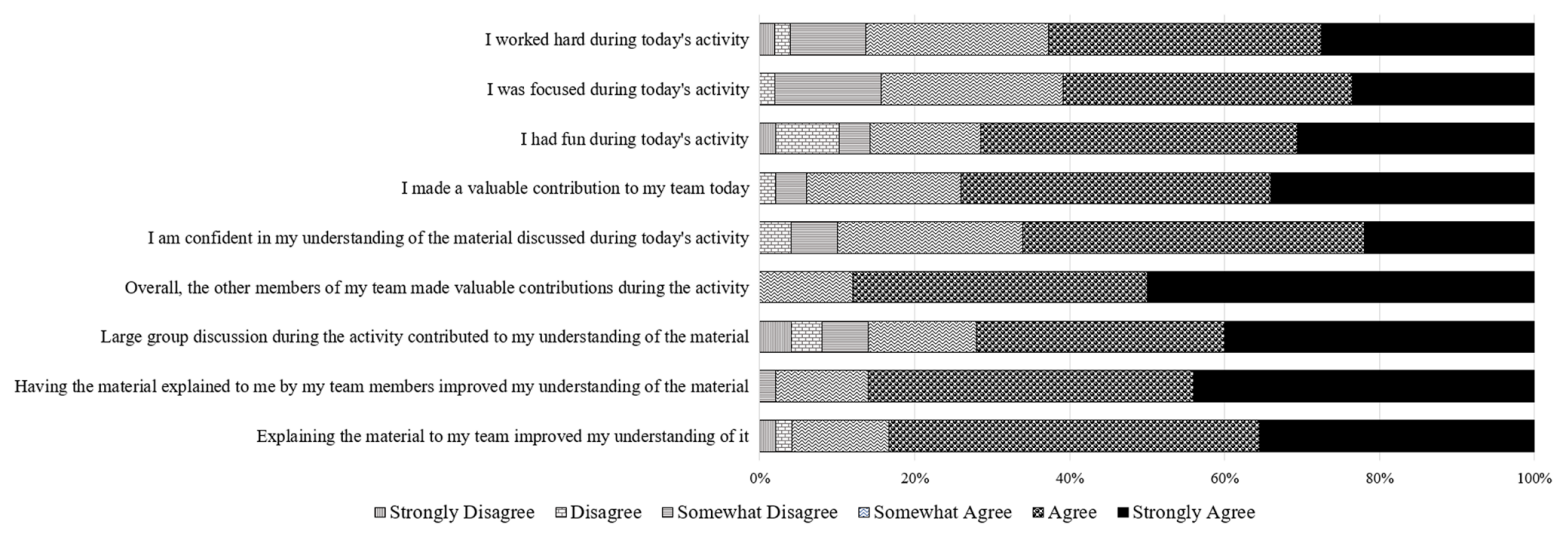
Student responses to post-session survey Likert scale questions assessing student perceptions of engagement.

Approximately 99% of the students completed the post-session survey, out of which 96% agreed or strongly agreed to being "confident in their understanding of the material discussed during the session.” Furthermore, for the prompts such as “team members made valuable contributions” and “team discussion improved their understanding of the material” were agreed or strongly agreed by majority of students ( Figure 1). Additionally, the students reported that they found the team-based approach useful because they received immediate instructor feedback.

Students displayed compelling engagement during the session and reported an increased understanding of the classification of COVID-19 variants and role of genetic mutations in viral pathogenesis ( Table 1). Another open-ended question asked the participants to “describe their feelings after the COVID-19 pandemic.” Many students expressed a feeling of being tired or exhausted by the pandemic and reported relief from the pandemic ( Table 2).

**Table 1.** Frequency of key themes from student (n=93) responses for the prompt: *“Describe how this TBL enhanced your understanding of the SARS-CoV-2 variants and COVID-19.”*. Some single entries by respondents had multiple key themes mentioned, and aggregated data is shown. Representative data from the Fall 2021 and Fall 2022 delivery of the TBL.

Describe how this TBL enhanced your understanding of SARS-CoV-2 variants and COVID-19.	# of students
Classification of variants	27
Role of genetic mutations with respect to variants	23
Understanding of the phylogenetic tree	16
General understanding of COVID-19	15
Transmissibility of the virus	14
Viral pathogenesis	7
Clarification of misconceptions	2

**Table 2.** Frequency of key themes from student (n=93) responses for the prompt: *“Describe your feelings after experiencing the COVID-19 pandemic.”*. Some single entries by respondents had multiple key themes mentioned, and aggregated data is shown. Representative data from the Fall 2021 and Fall 2022 delivery of the TBL.

Describe your feelings after experiencing the COVID-19 pandemic.	# of students
Feeling tired or exhausted	40
Relief that the pandemic is over	11
COVID-19 is still a critical issue	13
Gratitude for COVID-19 vaccines and research	11
Disappointed in the government response to the pandemic	3
More informed for future pandemics	3

## Discussion

Based on the academic climate during the COVID-19 pandemic, it has become imperative to develop sessions that directly approach healthcare students’ understanding and knowledge regarding any ongoing or recent infectious diseases impacting public health. The foundational sciences are well integrated into this TBL subject, enabling medical students to use higher-order virology and epidemiology concepts. Additionally, this framework allows room for revisions each year with updates in the most recent variants, or may also be expanded to other infectious diseases.

The students performed well when assessed summatively on the material. Following the session, the data analysis revealed an improvement in the knowledge and comprehension of COVID-19 and its variations. Students noted increased confidence in their understanding of COVID-19 variants. The open-ended responses produced favorable comments for the session, demonstrating similar trends in student involvement and learning. Many students mentioned how the lesson improved their capacity to decipher phylogenetic trees and understand variants of concern.

However, one of the limitations of this study is that due to the progressive updates to the global variant spread and change in different variant statuses, this framework needs to be updated until the day of delivery of the session, which may hinder the student’s ability to review the pre-reading material prior to the session. This can be resolved by ensuring that the pre-reading materials cover foundational knowledge and primary resources on viral variants, such as CDC or World Health Organization (WHO) informational pages.

For the future delivery of this framework, we anticipate expanding it with additional team application activities for any novel variants emerging from the phylogenic path of CoVs. In the future, we plan to adopt this framework as an Interprofessional Education (IPE) module for learners from multiple healthcare professional programs to promote diverse discussions. One of the most vital features of a framework’s success is the team-based delivery method.

## Conclusions

In conclusion, students’ perceptions of the session were positive each year, and team activities within the framework generated high levels of student engagement. Following the session, the students noted increased confidence in their understanding of COVID-19 variants. This TBL provides an opportunity to create an up-to-date platform for discussing novel and circulating COVID-19 variants within a framework that may be effectively amended periodically to capture current variants. Moreover, this can be adapted to an IPE setting by incorporating learners from multiple professional healthcare programmes.

## Disclosures

None to report.

## Ethics and consent

The study was approved by the Nova Southeastern University Institutional Review Board on September 17, 2020 (IRB Number 2021-203-NSU). The institutional review board deemed this study exempt from further review. Consent to complete the anonymous survey was provided by the participants at the beginning of the survey.

## Data Availability

Harvard Dataverse: An Active-Learning Framework for Educating Medical Students on SARS-CoV-2 Variants and COVID-19 Epidemiology. https://doi.org/10.7910/DVN/KDXL3F ( Chase & Prasad, 2024) This project contains the following underlying data:
An Active-Learning Framework for Educating Medical Students on SARS-CoV-2 Variants and COVID-19 Epidemiology.xlsx Harvard Dataverse: An Active-Learning Framework for Educating Medical Students on SARS-CoV-2 Variants and COVID-19 Epidemiology. https://doi.org/10.7910/DVN/KDXL3F ( Chase & Prasad, 2024) This project contains the following extended data:
An Active-Learning Framework for Educating Medical Students on SARS-CoV-2 Variants and COVID-19 Epidemiology_Pre-Post Survey Questions.docx Data are available under the terms of the Creative Commons Zero "No rights reserved" data waiver (CC0 1.0 Public domain dedication).

## References

[ref-1] Balwan S Fornari A DiMarzio P : Use of team-based learning pedagogy for internal medicine ambulatory resident teaching. *J Grad Med Educ.* 2015;7(4): 643–648. 10.4300/JGME-D-14-00790.1 26692979 PMC4675422

[ref-2] Beckler MD Chase AJ : Immune-mediated renal diseases: a team-based learning module for preclinical medical students. *MedEdPORTAL.* 2021;17: 11206. 10.15766/mep_2374-8265.11206 34970632 PMC8674152

[ref-3] Centers for Disease Control and Prevention: SARS-CoV-2 variant classifications and definitions. Updated Sept 2023. Accessed Nov 2021. Reference Source

[ref-4] Chase AJ Prasad S : An active-learning framework for educating medical students on SARS-CoV-2 variants and COVID-19 epidemiology. [Dataset], Harvard Dataverse, V1,2024. 10.7910/DVN/KDXL3F PMC11809145 39931307

[ref-5] De Figueiredo A Simas C Karafillakis E : Mapping global trends in vaccine confidence and investigating barriers to vaccine uptake: a large-scale retrospective temporal modelling study. *Lancet.* 2020;396(10255):898–908. 10.1016/S0140-6736(20)31558-0 32919524 PMC7607345

[ref-6] Fisher KA Nguyen N Fouayzi H : Impact of a physician recommendation on COVID-19 vaccination intent among vaccine hesitant individuals. *Patient Educ Couns.* 2023;106:107–112. 10.1016/j.pec.2022.09.013 36244947 PMC9523946

[ref-7] Kreps SE Goldfarb JL Brownstein JS : The relationship between US adults’ misconceptions about COVID-19 vaccines and vaccination preferences. *Vaccines (Basel).* 2021;9(8):901. 10.3390/vaccines9080901 34452025 PMC8402532

[ref-8] Kushner DB Pekosz A : Virology in the classroom: current approaches and challenges to undergraduate- and graduate-level virology education. *Annu Rev Virol.* 2021;8(1):537–558. 10.1146/annurev-virology-091919-080047 34242063

[ref-9] Mackenzie JS Smith DW : COVID-19: a novel zoonotic disease caused by a coronavirus from China: what we know and what we don't. *Microbiol Aust.* 2020; Ma20013. 10.1071/MA20013 32226946 PMC7086482

[ref-10] Mistry P Barmania F Mellet J : SARS-CoV-2 variants, vaccines, and host immunity. *Front Immunol.* 2022;12: 809244. 10.3389/fimmu.2021.809244 35046961 PMC8761766

[ref-11] Nayahangan LJ Konge L Russell L : Training and education of healthcare workers during viral epidemics: a systematic review. *BMJ Open.* 2021;11(5): e044111. 10.1136/bmjopen-2020-044111 34049907 PMC8166630

[ref-12] Neely SR Eldredge C Ersing R : Vaccine hesitancy and exposure to misinformation: a survey analysis. *J Gen Intern Med.* 2022;37(1):179–187. 10.1007/s11606-021-07171-z 34671900 PMC8528483

[ref-13] Office of the Surgeon General: Publications and reports of the Surgeon general. In: *Confronting Health Misinformation: The U.S. Surgeon General’s Advisory on Building a Healthy Information Environment*. US Department of Health and Human Services,2021. Reference Source 34283416

[ref-14] Opel DJ Lo B Peek ME : Addressing mistrust about COVID-19 vaccines among patients of color. *Ann Intern Med.* American College of Physicians,2021;174(5):698–700. 10.7326/M21-0055 33556271 PMC7888025

[ref-15] Parmelee D Michaelsen LK Cook S : Team-based learning: a practical guide: AMEE guide no. 65. *Med Teach.* 2012;34(5): e275–e287. 10.3109/0142159X.2012.651179 22471941

[ref-16] Plante JA Mitchell BM Plante KS : The variant gambit: COVID-19’s next move. *Cell Host Microbe.* 2021;29(4): 508–515. 10.1016/j.chom.2021.02.020 33789086 PMC7919536

[ref-17] Shahmanesh M Harling G Coltart CEM : From the micro to the macro to improve health: microorganism ecology and society in teaching infectious disease epidemiology. *Lancet Infect Dis.* 2020;20(6):e142–e147. 10.1016/S1473-3099(20)30136-5 32386611 PMC7252039

[ref-18] Wiggins BL Eddy SL Wener-Fligner L : ASPECT: a survey to Assess Student Perspective of engagement in an active-learning classroom. *CBE Life Sci Educ.* 2017;16(2): ar32. 10.1187/cbe.16-08-0244 28495936 PMC5459250

[ref-19] Zhu Z Lian X Su X : From SARS and MERS to COVID-19: a brief summary and comparison of severe acute respiratory infections caused by three highly pathogenic human coronaviruses. *Respir Res.* 2020;21(1): 224. 10.1186/s12931-020-01479-w 32854739 PMC7450684

