# Supplementary material for: An Active-Learning Framework for Educating Medical Students on SARS-CoV-2 Variants and COVID-19 Epidemiology
Source: MedEdPublish (2016). 2024 Nov 15;14:279. [Version 1] doi: 10.12688/mep.20540.1 (PMC11809145; doi:10.12688/mep.20540.1)
Supplement: Peer review history

# Reviewer report: Reviewer response for version 1

**DOI:** 10.21956/mep.21985.r40665

**Published:** 2025-01-07

**Authors:** Syeda Sadia Fatima

**Affiliations:** 1 Aga Khan University, Karachi, Pakistan

This authors presents a comprehensive approach to a team-based learning (TBL) module developed for first-year medical students. The TBL module was integrated into the Hematology Block and focused on understanding the emergence and transmission dynamics of SARS-CoV-2 variants. 

 Results indicate a significant level of student engagement, with high post-session survey completion rates and positive feedback on understanding key concepts. Students reported increased confidence in their comprehension of SARS-CoV-2 variants, with many highlighting the benefits of team discussions and immediate feedback from instructors.

**There a few areas that the authors may consider addressing to improve the manuscript even further: **

- Include a discussion on the method used for thematic analysis of open-ended survey responses.
- Provide more details on how common misconceptions were addressed during the large-group discussion.
- Discuss the potential for bias in student feedback and consider including a pre-post knowledge assessment or control group for future iterations.
- Clarify the nature and scope of instructor feedback during team application activities.
- Add a brief description of how themes in Tables 1 and 2 were identified and categorized.

Is the rationale for developing the new method (or application) clearly explained?

Yes

Is the description of the method technically sound?

Yes

Are the conclusions about the method and its performance adequately supported by the findings presented in the article?

Yes

If any results are presented, are all the source data underlying the results available to ensure full reproducibility?

Yes

Are sufficient details provided to allow replication of the method development and its use by others?

Yes

Reviewer Expertise:

Technology enhanced education

I confirm that I have read this submission and believe that I have an appropriate level of expertise to confirm that it is of an acceptable scientific standard.

# Reviewer report: Reviewer response for version 1

**DOI:** 10.21956/mep.21985.r40666

**Published:** 2025-01-04

**Authors:** Forman Erwin Siagian

**Affiliations:** 1 Department of Parasitology, Faculty of Medicine, Universitas Kristen Indonesia, East Jakarta, Special Capital Region of Jakarta, Indonesia

This manuscript, presented by Dr Prasad and Dr Chase, offers an insightful exploration of the role of incorporating a newly develop module as a quick response to the global emerging disease into ongoing medical curricula. By doing so, the academic team exposed a foundational concept to certain clinical problem and prepared students of professional healthcare programs benefit from being adept with every aspects of the evolution and spread of SARS-CoV-2 variants, which focus on transmission and emergence dynamics of viral variants among future healthcare providers through an engaging framework; which further enrich their understanding regarding patient concerns and building their future professionalism.  

 When I was asked to review this article, I reflected on the novelty (Elston, 2021) of this manuscript brings to the field of medicine, especially medical education. To my opinion, this manuscript is scientifically vigorous and scholarly appropriate. What is important to note is that the respondents found the team-based approach useful for them because they received immediate instructor feedback and increased their understanding. Even though, traditional approach in medical education such as lectures, tutorials, practicum and laboratory classes still play an important role in teaching. Even with the growing influence of personalized learning experience facilitated by artificial intelligence (Mir *et al,* 2023), these authors emphasize the irreplaceable value of novelty and adaptation in a truly blended learning of continuing medical education.

 I appreciate how the manuscript keeps a balance between the promises and limitations of this new method, particularly highlighting the importance of active learning of any ongoing or recent infectious diseases impacting global public health, and also enable the authority regarding the possibility of revision and expansion to other infectious disease. However, a more detailed analysis of how this new method could complement rather than compete with traditional class based, teacher centered (single direction) experiences could have strengthened the discussion. Overall, I find the manuscript a positive contribution to the ongoing improvement in medical education.

 In this unstable aging world, disease outbreaks may come and go, socioeconomic conditions can rise and fall, society is always the most vulnerable in terms of health, but the spirit and noble values ​​of education (El Bingawi & Haleem, 2023), including medical education, are a torch of light about human resilience and fighting power in adapting to threats and challenges with the noblest aim of making daily human life and humanity better and more equitable.

Is the rationale for developing the new method (or application) clearly explained?

Yes

Is the description of the method technically sound?

Yes

Are the conclusions about the method and its performance adequately supported by the findings presented in the article?

Yes

If any results are presented, are all the source data underlying the results available to ensure full reproducibility?

Yes

Are sufficient details provided to allow replication of the method development and its use by others?

Yes

Reviewer Expertise:

Parasitology, Mycology, Medical Education

I confirm that I have read this submission and believe that I have an appropriate level of expertise to confirm that it is of an acceptable scientific standard.

## References

[1] : The novelty effect. *Journal of the American Academy of Dermatology* .2021;85(3) : 10.1016/j.jaad.2021.06.846 565-566 10.1016/j.jaad.2021.06.846 34153390

[2] : Application of Artificial Intelligence in Medical Education: Current Scenario and Future Perspectives. *J Adv Med Educ Prof* .2023;11(3) : 10.30476/JAMP.2023.98655.1803 133-140 10.30476/JAMP.2023.98655.1803 37469385 PMC10352669

[3] : A palpable change in medical education post-COVID-19 pandemic. *Pan Afr Med J* .2023;46: 10.11604/pamj.2023.46.70.37676 70 10.11604/pamj.2023.46.70.37676 38282774 PMC10822105

# Reviewer report: Reviewer response for version 1

**DOI:** 10.21956/mep.21985.r40128

**Published:** 2025-01-02

**Authors:** Ahmed Msherghi

**Affiliations:** 1 Neuroradiology, The University of Texas MD Anderson Cancer Center Department, Houston, Texas, USA

This quality improvement study with pre and post-training feedback aimed to teach early-stage medical students about the virology and epidemiology aspects of COVID-19.

 The results flow is well organized, and the method is sufficiently described.

 I would suggest you add the full names of iRAT and tRAT early in the method or the results and in the abstract section, as many readers will not have prior background about what they mean.

 Also, please provide details of the duration of the studies and how many groups yielded each year in the method section instead of briefly mentioning them in the result section.

Is the rationale for developing the new method (or application) clearly explained?

Yes

Is the description of the method technically sound?

Yes

Are the conclusions about the method and its performance adequately supported by the findings presented in the article?

Partly

If any results are presented, are all the source data underlying the results available to ensure full reproducibility?

Yes

Are sufficient details provided to allow replication of the method development and its use by others?

Yes

Reviewer Expertise:

teaching and learning improvement, public health and statistical and professional writing.

I confirm that I have read this submission and believe that I have an appropriate level of expertise to confirm that it is of an acceptable scientific standard.

# Reviewer report: Reviewer response for version 1

**DOI:** 10.21956/mep.21985.r40119

**Published:** 2024-12-10

**Authors:** Varna Taranikanti

**Affiliations:** 1 Oakland University William Beaumont School of Medicine, Oakland, USA

Thank you for inviting me to review the article titled" An Active-Learning Framework for Educating Medical Students on SARS-CoV-2 Variants and COVID-19 Epidemiology".

 The authors have done a commendable job in  implementation of the team-based learning (TBL) framework in response to the COVID-19 pandemic into medical education. It indeed is helpful for medical students to apply foundational concepts in understanding clinical problems through individual and teamwork while receiving immediate feedback.

**Introduction:** The introduction was well written with adequate review of literature.  The educational objectives are clear however, the rationale for developing this method could be made more clear to understand the learning outcomes from the TBL framework.

**Methodology: **The increase in scores from the iRAT to the tRAT is a positive outcome; however, more detail on the assessment methods and the types of questions asked would provide insight into how well the students truly understood the material. For instance, were the questions primarily factual, or did they require application and synthesis of knowledge? Examples of the questions asked as a supplement would help.

**Results: ** The evaluation primarily focuses on immediate post-session outcomes. It would be beneficial to assess long-term retention of this knowledge through follow-up assessments or surveys in later semesters to determine the lasting impact of the TBL framework.

**Discussion:** The authors mention about integration of different disciplines from foundational sciences. However, providing specific examples such as integration of virology and epidemiology as applied during the session would enhance clarity.

 The authors  mention about improvement in knowledge and comprehension. Were specific assessments or evaluation tools employed to measure comprehension beyond self-reported confidence?

**Conclusion:** The author’s focus was on student engagement and understanding.  However, a broader perspective on how this TBL framework contributes to public health education could enhance the conclusion.

 The conclusion summarizes the students' positive perceptions effectively. However, including quantitative data or specific feedback examples could provide stronger evidence to support these claims.

 The adaptability of the TBL framework is a key strength of this article; however, it could be reinforced by discussing potential challenges to implementing in other infectious diseases or IPE elements.  The authors could also mention about  plans for future adaptations. What steps will be taken to ensure successful implementation of the expanded activities and IPE components.

**Discussion and Conclusion:**  The authors have done a good job  in this D&C section and  provided valuable insights into the effectiveness of the TBL framework for this important topic however, enhancing clarity, detail, and depth in certain areas could strengthen the overall analysis and presentation of the findings.

Is the rationale for developing the new method (or application) clearly explained?

Partly

Is the description of the method technically sound?

Yes

Are the conclusions about the method and its performance adequately supported by the findings presented in the article?

Yes

If any results are presented, are all the source data underlying the results available to ensure full reproducibility?

Yes

Are sufficient details provided to allow replication of the method development and its use by others?

Partly

Reviewer Expertise:

Medical Education, TBL, SDL, AI in medical education, Curriculum integration

I confirm that I have read this submission and believe that I have an appropriate level of expertise to confirm that it is of an acceptable scientific standard.
